# Risk factors for hepatocellular carcinoma rupture: multicentre retrospective study

**DOI:** 10.1093/bjsopen/zraf105

**Published:** 2025-11-04

**Authors:** Feng Xia, Yiyang Liu, Hongwei Huang, Xulin Liu, Jing Yan, Zhancheng Qiu, Qiao Zhang, Zhenheng Wu, Zhiyuan Huang, Renjie Wei, Li Lin, Liping Liu, Shuangqin Han, Yulin Yuan, Huaxuan Yin, Guobing Xia, Yunyan Wan, Shuo Xiao, Guoxiang Zhou, Xiafei Xia, Huapeng Sun, Shuai Wang, Jun Zheng, Hengyi Gao, Jiang Zheng, Li Ren, Ali Mo, Lin Ye, Shun Ruan, Xiaoping Chen, Qi Cheng, Bixiang Zhang, Peng Zhu

**Affiliations:** Department of Hepatic Surgery, Tongji Hospital, Tongji Medical College of Huazhong University of Science and Technology, Wuhan, China; Department of Hepatic Surgery, Tongji Hospital, Tongji Medical College of Huazhong University of Science and Technology, Wuhan, China; Department of Hepatobiliary Surgery, Union Hospital, Tongji Medical College, Huazhong University of Science and Technology, Wuhan, China; Department of Hepatic Surgery, Tongji Hospital, Tongji Medical College of Huazhong University of Science and Technology, Wuhan, China; Department of Hepatic Surgery, Tongji Hospital, Tongji Medical College of Huazhong University of Science and Technology, Wuhan, China; Department of Ultrasound in Medicine, Second Affiliated Hospital of Zhejiang University School of Medicine, Zhejiang, China; Department of General Surgery, West China Hospital, Sichuan University, Chengdu, China; Department of Hepatic Surgery, Zhongshan People's Hospital Affiliated to Guangdong Medical University, Zhongshan, China; Department of Hepatopancreatobiliary Surgery, First Affiliated Hospital of Fujian Medical University, Fuzhou, China; Department of General Surgery, General Hospital of Central Theater Command, Wuhan, China; Department of Neurosurgery, Zhongnan Hospital of Wuhan University, Wuhan, China; Clinic Center of Human Gene Research, Union Hospital, Tongji Medical College, Huazhong University of Science and Technology, Wuhan, China; Department of Hepatobiliary Surgery, Shenzhen People's Hospital, Shenzhen, China; Department of Hepatobiliary Surgery, Tianjin First Central Hospital, Tianjin Medical University, Tianjin, China; School of Medicine, Wuhan University of Science and Technology, Wuhan, China; Department of Hepatic-Biliary-Pancreatic Surgery, First People's Hospital of Foshan, Foshan, China; Department of Hepatobiliary and Pancreatic Surgery, Huangshi Central Hospital, Hubei Polytechnic University, Huangshi, China; Department of Hepatobiliary Pancreatic Surgery, Taihe Hospital, Shiyan, China; Department of Cardiac Surgery, Guangdong Provincial People’s Hospital, Southern Medical University, Guangzhou, China; Department of Heart Center, Women and Children's Hospital of Qingdao University, Qingdao, China; Department of Organ Transplantation, First Affiliated Hospital of Kunming Medical University, Kunming Medical University, Kunming, China; Department of General Surgery, Xiangyang Central Hospital, Affiliated Hospital of Hubei University of Arts and Science, Xiangyang, China; Department of Hepatobiliary Surgery, Jingzhou Central Hospital, Jingzhou, China; Department of Science and Education, Shenzhen Baoan District People's Hospital, Guangdong, China; Department of Hepatobiliary and Pancreatic Surgery, Shenzhen Longhua District People's Hospital, Guangdong, China; Department of Cardiology, Wuhan Yaxin General Hospital Affiliated to Wuhan University of Science and Technology, Wuhan, China; Department of Hepatobiliary Surgery, Affiliated Hospital of Qinghai University, Xining, China; Department of General Surgery, First Affiliated Hospital of Shaoyang University, Shaoyang, China; Department of Hepatobiliary and Pancreatic Surgery, Affiliated Hospital of Guilin Medical University, Guilin, China; Department of Surgery, Dongguan Hepatobiliary Hospital, Dongguan, China; Department of Hepatic Surgery, Tongji Hospital, Tongji Medical College of Huazhong University of Science and Technology, Wuhan, China; Department of Hepatic Surgery, Tongji Hospital, Tongji Medical College of Huazhong University of Science and Technology, Wuhan, China; Department of Hepatic Surgery, Tongji Hospital, Tongji Medical College of Huazhong University of Science and Technology, Wuhan, China; Department of Hepatic Surgery, Tongji Hospital, Tongji Medical College of Huazhong University of Science and Technology, Wuhan, China

**Keywords:** tumour rupture, prediction model, machine learning

## Abstract

**Background:**

Hepatocellular carcinoma (HCC) rupture is a life-threatening complication associated with poor prognosis. This study comprehensively analysed risk factors for HCC rupture and developed a predictive model supplemented by machine learning models for early risk identification and clinical decision-making.

**Methods:**

This retrospective study analysed patients with and without HCC rupture from tertiary centres in China between January 2016 and June 2019. Propensity score matching (PSM) was used to reduce baseline differences between the rupture and non-rupture groups. Random forest and deep learning models were developed to enhance predictive accuracy and interpret variable importance. Model performance was evaluated using metrics such as precision, recall, and the F1 score across training, validation, and test cohorts.

**Results:**

Among the 5952 HCC patients, the median follow-up duration was 48.6 months. Key risk factors for HCC rupture identified in this study include cirrhosis, protrusion ratio, and tumour maximum length. The CAPTure nomogram, constructed based on these predictors, yielded area under the curve (AUC) values of 0.857, 0.824, and 0.840 in the training, validation, and test cohorts, respectively. In the test cohort, the random forest and deep learning models achieved AUCs of 0.870 and 0.872, respectively.

**Conclusion:**

This study provides a comprehensive analysis of risk factors for HCC rupture and introduces the CAPTure model as a practical and accurate tool for clinical use. By integrating traditional and machine learning approaches, the findings of this study offer robust methods for early risk assessment, resource optimization, and improved management of HCC rupture.

## Teaser

This study comprehensively analysed risk factors for hepatocellular carcinoma rupture in a nationwide Chinese cohort and developed the CAPTure (Cirrhosis, Assessment of Protrusion ratio, and Tumour maximum length) predictive model. The model demonstrated strong predictive accuracy (area under the curve 0.857–0.840) across training, validation, and test cohorts. The inclusion of machine learning techniques further enhanced prediction capabilities, emphasizing early risk identification and personalized interventions to improve clinical outcomes.

## Introduction

Hepatocellular carcinoma (HCC) is a leading cause of cancer-related mortality worldwide, with particularly high incidence rates in Asia^1,2^. Among its life-threatening complications, spontaneous tumour rupture poses a serious clinical challenge, often leading to haemorrhagic shock, tumour dissemination, and a significant decrease in overall survival (OS)^3–5^. Reported HCC rupture rates in Asian populations range from 10 to 26%, and the associated acute-phase mortality can reach 75%, underscoring the urgent need for early risk identification and preventive strategies in this high-risk population^6–9^.

Despite these findings, many studies on risk factors for HCC rupture remain descriptive, focusing on qualitative assessments such as whether tumours protrude beyond the liver surface. For a severe complication like tumour rupture, the lack of precise quantitative metrics not only limits the early identification of high-risk patients but also hampers the development of effective prevention and intervention strategies. Thus, transforming these qualitative features into quantifiable imaging-based indicators, combined with easy-to-use predictive tools, is of paramount importance. These measures may not only reduce the incidence of rupture but also offer patients hope for prolonged survival.

The aims of the present study were to comprehensively analyse rupture-associated risk factors using a nationwide data set of patients from 26 tertiary centres across China and to develop the CAPTure (Cirrhosis, Assessment of Protrusion ratio, and Tumour maximum length) model for predicting the risk of spontaneous HCC rupture.

## Methods

### Patients

**Fig. 1:**
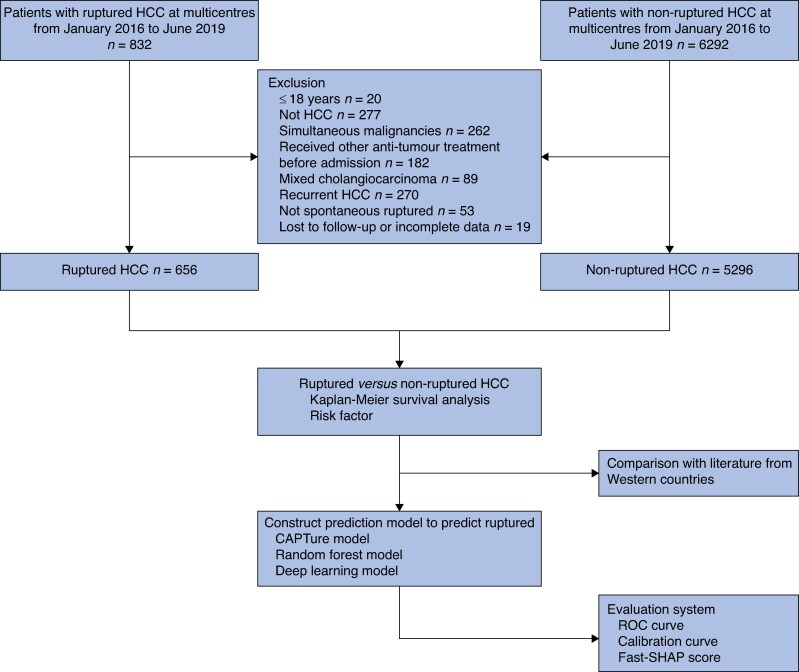
Flow chart for the inclusion and exclusion of patients with non-ruptured and ruptured HCC in nationwide multicentre cohorts HCC, hepatocellular carcinoma; ROC, receiver operating characteristic; CAPTure, Cirrhosis, Assessment of Protrusion ratio, and Tumour maximum length; FastSHAP, efficient and model-agnostic approximation of SHapley Additive exPlanations.

Patients were eligible for inclusion in the study if they were: aged ≥ 18 years; had a diagnosis of HCC either pathologically confirmed or established based on imaging combined with serum tumour markers, according to accepted diagnostic guidelines^10^; and newly diagnosed (first-time diagnosis), with no history of HCC. Only patients who underwent contrast-enhanced computed tomography (CT) as part of their initial diagnostic evaluation were included in the study, because this imaging modality was essential for determining rupture status. The exclusion criteria were the presence of simultaneous malignancies, treatment with antitumour therapies before admission to hospital, a diagnosis of mixed cholangiocarcinoma, recurrent HCC, HCC rupture that was not spontaneous, and incomplete clinical or follow-up data (*Fig. 1*).

This study was conducted in accordance with both the Declaration of Helsinki and the Declaration of Istanbul. The study protocol was approved by the Ethics Review Board of Tongji Hospital (Wuhan, China). Written informed consent was provided by all patients.

### Imaging assessment

All patients underwent cross-sectional imaging assessments conducted collaboratively by two senior radiologists and one hepatic surgeon. For the diagnosis and evaluation of tumour rupture, contrast-enhanced CT was used as the primary and standardized imaging modality, owing to its high temporal resolution and superior sensitivity for detecting acute haemorrhagic events.

The CT criteria used to define the tumour rupture were high-attenuation perihepatic fluid or haemoperitoneum, disruption of the tumour capsule or an irregular tumour margin extending beyond the liver surface, contrast extravasation in the arterial phase, or the presence of peritumoural haematoma.

In addition to imaging, intraoperative confirmation or clinical symptoms (for example, acute abdominal pain with hypotension and imaging-confirmed bleeding source) were considered supportive but not sufficient alone for diagnosis. Only patients that fulfilled CT-based imaging criteria were classified as having radiologically confirmed tumour rupture. To ensure diagnostic consistency and real-time reflection of tumour status, only contrast-enhanced CT scans performed within 24 hours of hospital admission were included in the analysis.

### Management of patients with ruptured and non-ruptured HCC

All HCC patients, including those with tumour rupture, received treatment tailored to their clinical condition and tumour characteristics. For patients with haemodynamic instability due to acute abdominal bleeding, transarterial embolization or transarterial chemoembolization (TACE) was the preferred initial treatment. Conservative management was reserved for select patients when interventions were deemed unnecessary or unfeasible.

For patients with resectable tumours and preserved liver function, surgery was performed once bleeding was controlled and haemodynamic stability was achieved after initial treatment. The aim of emergency surgery for patients with a ruptured tumour was to achieve curative resection and haemostasis. For patients who did not require immediate surgery, staged delayed hepatectomy was considered after comprehensive evaluation. For patients without ruptured HCC, treatment strategies included liver resection, TACE, or systemic therapies depending on tumour burden, liver function, and patient preferences.

All patients underwent thorough assessments, including imaging evaluations (abdominal ultrasound, enhanced CT, or magnetic resonance imaging), cardiopulmonary and renal function assessments, serological tests, and liver function scoring (for example, Model for End-Stage Liver Disease (MELD) score, Child–Pugh score, and the albumin–bilirubin (ALBI) score). The optimal management strategy for each patient was determined by a multidisciplinary team.

### Radiological measurement of protrusion ratio

The protrusion ratio was defined as the maximum vertical distance from the outermost edge of the tumour extending beyond the liver capsule to the adjacent liver surface, divided by the tumour's maximum diameter. Measurements were performed on axial contrast-enhanced CT images by two senior radiologists using multiplanar reconstruction. Interobserver consistency was assessed, and disagreements were resolved by joint review. A protrusion ratio cut-off value of 0.2 was used to categorize tumours with high protrusion risk, which was determined using the Youden index for receiver operating characteristic (ROC) curve analysis in the training cohort.

Details regarding management, model construction, follow-up, and data analysis are provided in the *supplementary methods*.

### Statistical analysis

Continuous variables are presented as the median (interquartile range), whereas categorical variables are presented as frequencies with percentages. Categorical variables were compared using χ^2^ tests or Fisher's exact tests. Univariate and multivariate logistic regression analyses were conducted to identify factors associated with tumour rupture. Kaplan–Meier curves with log-rank tests were used to analyse overall survival. Univariate and multivariate Cox regression analyses were performed to identify factors influencing overall survival. Variables with *P* < 0.05 in the univariate analysis were included in the multivariate regression analysis. Feature importance in the machine learning models was evaluated using FastSHAP, an efficient and model-agnostic approximation of SHapley Additive exPlanations (SHAP). FastSHAP accelerates SHAP value estimation by training a surrogate explainer model, providing interpretable insights into each variable’s contribution to model predictions, while maintaining high computational efficiency^11^.

Variable selection and modelling were conducted using logistic regression. The nomogram was developed, validated, and tested using R version 4.1.2 (R Foundation for Statistical Computing, Vienna, Austria). Random forest and deep learning models were built, validated, and tested using Python version 3.7.6 (Python Software Foundation, Wilmington, DE, USA). Hyperparameter tuning and performance evaluation of the models were also conducted in Python.

Evaluation metrics for the machine learning models and nomogram, including ROC curves, calibration curves, and FastSHAP scores, were generated using R software. Kaplan–Meier curves and ROC curves were also plotted using R. All statistical analyses were performed using SPSS^®^ version 25.0 (IBM, Armonk, NY, USA). Two-tailed *P* < 0.05 was considered statistically significant.

## Results

### Comparison of baseline characteristics between patients with and without HCC rupture

Baseline characteristics are summarized in *Table 1*. Of the 5952 patients included in the study, 656 were classified as having ruptured HCC and 5296 were classified as having non-ruptured HCC. Compared with the non-ruptured HCC group, the ruptured HCC group had a higher proportion of large (> 5 cm) tumours (2610 (49.3%) *versus* 458 (69.8%); *P* < 0.001), tumours protruding beyond the liver surface (2358 (44.5%) *versus* 494 (75.3%); *P* < 0.001), and cirrhosis (3008 (56.8%) *versus* 556 (84.8%); *P* < 0.001), as well as a higher mean(standard deviation) protrusion ratio (0.12(0.04) *versus* 0.27(0.06); *P* < 0.001). Tumours located in the left lobe were more frequent in the ruptured than non-ruptured group (275 (41.9%) *versus* 1249 (23.6%); *P* < 0.001), and the ruptured group had a higher prevalence of sarcopenia (374 (57.0%) *versus* 2462 (46.5%); *P* < 0.001). Within the ruptured HCC group, a higher proportion of patients had hypertension and a history of alcohol consumption (*P* < 0.001 and *P* = 0.003, respectively). The proportion of patients positive for hepatitis B surface antigen (HBsAg) was significantly higher in the ruptured than non-ruptured group (602 (91.8%) *versus* 4316 (81.5%); *P* < 0.001; *Table 1*).

**Table 1:** Baseline characteristics of patients with ruptured and non-ruptured hepatocellular carcinoma (*n* = 5952 overall)

	HCC ruptured (*n* = 656)	HCC not ruptured (*n* = 5296)	*P*‡
**Sex**			0.494
Male	558 (85.1%)	4450 (84.0%)	
Female	98 (14.9%)	846 (16.0%)	
**Age**			0.919
≤ 60 years	500 (76.2%)	4046 (76.4%)	
> 60 years	156 (23.8%)	1250 (23.6%)	
**BMI**			0.638
≤ 30 kg/m^2^	611 (93.1%)	4958 (93.6%)	
> 30 kg/m^2^	45 (6.9%)	338 (6.4%)	
**Tumour maximum length**			<0.001
≤ 5 cm	198 (30.2%)	2686 (50.7%)	
> 5 cm	458 (69.8%)	2610 (49.3%)	
**Protrusion from the liver surface**			<0.001
No	162 (24.7%)	2938 (55.5%)	
Yes	494 (75.3%)	2358 (44.5%)	
Protrusion ratio, mean(s.d.)	0.27(0.06)	0.12(0.04)	<0.001
**No. of tumours**			0.639
Single	494 (75.3%)	4032 (76.1%)	
Multiple	162 (24.7%)	1264 (23.9%)	
**Tumour location**			<0.001
Left lobe	275 (41.9%)	1249 (23.6%)	
Right lobe	301 (45.9%)	3362 (63.5%)	
Both	80 (12.2%)	685 (12.9%)	
**AFP**			0.778
≤ 400 ng/ml	316 (48.2%)	2582 (48.8%)	
> 400 ng/ml	340 (51.8%)	2714 (51.2%)	
**Cirrhosis**			<0.001
No	100 (15.2%)	2288 (43.2%)	
Yes	556 (84.8%)	3008 (56.8%)	
Cause: HBV	507 (91.2%)‡	2765 (91.9%)‡	
Cause: alcohol	45 (8.1%)‡	235 (7.8%)‡	
Cause: other§	4 (0.7%)‡	8 (0.2%)‡	
**CSPH**			0.814
No	484 (73.8%)	3930 (74.2%)	
Yes	172 (26.2%)	1366 (25.8%)	
**Child–Pugh grade**			0.801
A	396 (60.4%)	3224 (60.9%)	
B	260 (39.6%)	2072 (39.1%)	
MELD score, median (i.q.r.)	10.2 (8.5–13.2)	9.0 (7.6–11.5)	0.045
**PVTT**			<0.001
Present	196 (29.9%)	370 (7.0%)	
Absent	460 (70.1%)	4926 (93.0%)	
**HBsAg**			<0.001
No	54 (8.2%)	980 (18.5%)	
Yes	602 (91.8%)	4316 (81.5%)	
**HCV‡**			0.218
No	654 (99.7%)	5290 (99.9%)	
Yes	2 (0.3%)	6 (0.1%)	
**Drinking history**			0.003
No	454 (69.2%)	4058 (76.6%)	
Yes	202 (30.8%)	1238 (23.4%)	
**Hypertension**			<0.001
No	471 (71.8%)	4126 (77.9%)	
Yes	185 (28.2%)	1170 (22.1%)	
**Sarcopenia**			<0.001
No	282 (43.0%)	2834 (53.5%)	
Yes	374 (57.0%)	2462 (46.5%)	
**Albumin**			0.359
≤ 35 g/L	298 (45.4%)	2306 (43.5%)	
> 35 g/L	358 (54.6%)	2990 (56.5%)	
**ALBI score**			0.253
1	596 (90.9%)	4832 (91.2%)	
2	51 (7.8%)	424 (8.0%)	
3	9 (1.4%)	40 (0.8%)	
**Platelet count**			0.059
≤ 150x10³/µL	138 (21.0%)	954 (18.0%)	
> 150x10³/µL	518 (79.0%)	4342 (82.0%)	
**Creatinine**			0.433
≤ 1.2 mg/dl	648 (98.8%)	5248 (99.1%)	
> 1.2 mg/dl	8 (1.2%)	48 (0.9%)	
**Bilirubin**			0.749
≤ 1.2 mg/dl	641 (97.7%)	5185 (97.9%)	
> 1.2 mg/dl	15 (2.3%)	111 (2.1%)	

Values are *n* (%) unless otherwise stated. *χ^2^ test with Yates correction. †Fisher's exact test. ‡Proportion of patients with liver cirrhosis. §‘Other’ refers to liver cirrhosis caused by autoimmune factors, genetic and metabolic diseases, fatty liver. HCC, hepatocellular carcinoma; BMI, body mass index; s.d., standard deviation; AFP, α-fetoprotein; HBV, hepatitis B virus; CSPH, clinically significant portal hypertension; MELD, Model for End-Stage Liver Disease; i.q.r., interquartile range; PVTT, portal vein tumour thrombus; HBsAg, hepatitis B surface antigen; HCV, hepatitis C virus; ALBI, albumin–bilirubin.

After 1:1 propensity score matching, baseline characteristics between the ruptured and non-ruptured groups were well balanced across key clinical variables, confirming the comparability of the two cohorts (*Table S1*). Univariate and multivariate Cox regression analyses indicated that rupture was a significant risk factor for overall survival in HCC patients, with hazard ratios (HRs) of 2.451 (95% confidence interval (c.i.) 2.222 to 2.893; *P* < 0.001) and 1.966 (95% c.i. 1.573 to 2.456; *P* < 0.001; *Table 2*). Kaplan–Meier analysis after propensity score matching confirmed that overall survival remained significantly worse in the ruptured than non-ruptured group (HR 2.36; 95% c.i. 2.06 to 2.72; *P* < 0.001; *Fig. S2*). In a subgroup analysis restricted to patients who underwent one-stage hepatectomy, overall survival remained significantly worse in the ruptured than non-ruptured group (HR 2.28; 95% c.i. 1.99 to 2.62; *P* < 0.001; *Fig. S3*), suggesting that tumour rupture independently contributes to poorer outcomes, even among surgically treated patients.

**Table 2:** Univariate and multivariate Cox regression analysis of factors affecting overall survival in the entire hepatocellular carcinoma cohort

	Univariate analysis		Multivariate analysis	
	HR*	*P*	HR*	*P*
**Sex**		0.120		
Male	Reference			
Female	1.501 (0.899, 2.504)			
**Age (years)**		0.062		
≤ 60	Reference			
> 60	1.437 (0.883, 1.906)			
**BMI (kg/m^2^)**		0.391		
≤ 30	Reference			
> 30	0.926 (0.764, 1.544)			
**Tumour maximum length (cm)**		0.023		0.016
≤ 5	Reference		Reference	
> 5	1.486 (1.056, 2.093)		1.399 (1.047, 1.971)	
**No. of tumours**		0.191		
Single	Reference			
Multiple	2.150 (0.683, 6.774)			
**Tumour location**		0.552		
Left lobe	1.126 (0.694, 1.522)			
Right lobe	Reference			
Both	1.094 (0.716, 1.426)			
**AFP (ng/ml)**		<0.001		<0.001
≤ 400	Reference		Reference	
> 400	1.809 (1.358, 2.410)		1.752 (1.340, 2.449)	
**Cirrhosis**		0.520		
No	Reference			
Cause: HBV	1.059 (0.889, 1.262)	0.149		
Cause: alcohol	1.162 (0.775, 1.615)	0.628		
Cause: other	1.071 (0.815, 1.299)	0.284		
**CSPH**		<0.001		<0.001
No	Reference		Reference	
Yes	1.575 (1.269, 1.904)		1.487 (1.204, 1.753)	
**Child–Pugh grade**		<0.001		<0.001
A	Reference		Reference	
B	2.018 (1.350, 3.015)		1.913 (1.209, 2.970)	
**PVTT**		<0.001		<0.001
No	Reference		Reference	
Yes	2.001 (1.602, 2.500)		1.845 (1.377, 2.291)	
**HBsAg**		0.351		
No	Reference			
Yes	1.247 (0.765, 1.584)			
**HCV**		0.224		
No	Reference			
Yes	1.075 (0.910, 1.208)			
**Drinking history**		0.460		
No	Reference			
Yes	1.122 (0.827, 1.522)			
**Sarcopenia**		<0.001		<0.001
No	Reference		Reference	
Yes	1.698 (1.214, 2.347)		1.576 (1.216, 1.954)	
**Hypertension**		0.330		
No	Reference			
Yes	1.197 (0.825, 1.671)			
**Albumin (g/L)**		0.314		
≤ 35	Reference			
> 35	0.876 (0.571, 1.377)			
**ALBI score**		0.022		
1	Reference			
2	1.205 (1.105, 1.416)			
3	1.378 (1.164, 1.588)			
**Platelet count (x10³/µL)**		0.296		
≤ 150	Reference			
> 150	0.866 (0.749, 1.133)			
**Creatinine (mg/dl)**		0.339		
≤ 1.2	Reference			
> 1.2	1.276 (0.796, 1.690)			
**Bilirubin (mg/dl)**		0.480		
≤ 1.2	Reference			
> 1.2	1.189 (0.682, 1.459)			
**Rupture**		<0.001		<0.001
No	Reference		Reference	
Yes	2.451 (2.222, 2.893)		1.966 (1.573, 2.456)	

^*^Values in parentheses are 95% confidence intervals. HR, hazard ratio; BMI, body mass index; AFP, α-fetoprotein; HBV, hepatitis B virus; CSPH, clinically significant portal hypertension; MELD, Model for End-stage Liver Disease; PVTT, portal vein tumour thrombus; HBsAg, hepatitis B surface antigen; HCV, hepatitis C virus; ALBI, albumin–bilirubin.

### Baseline characteristics across the training, validation, and test cohorts and univariate and multivariate logistic regression analysis of risk factors for rupture

Of the 5952 patients, 4166 (70.0%) were assigned to the training cohort, 892 (15.0%) were assigned to the validation cohort, and 894 (15.0%) were assigned to the test cohort. Baseline characteristics were well balanced across the three cohorts, including variables sex, age, body mass index, tumour size, tumour protrusion beyond the liver surface, tumour location, cirrhosis, α-fetoprotein levels, portal vein tumour thrombus (PVTT), and HbsAg status.

There were no significant differences in the proportion of tumours > 5 cm, protrusion ratio, and location in the right lobe among the three cohorts. In addition, indicators of baseline liver function (Child–Pugh grade, albumin, alanine aminotransferase, aspartate aminotransferase, alkaline phosphatase, γ-glutamyl transferase) and general conditions (such as sarcopenia and hypertension) were comparable among the three cohorts, ensuring model comparability and reliability for subsequent analyses (*Table S2*).

Using the Youden index, the optimal cut-off for the protrusion ratio was determined to be 0.2051 and was set at 0.2 (*Fig. S4*). Univariable and multivariable logistic regression analyses were conducted using the training cohort (*Table 3*). Variables with *P* < 0.05 in univariate analysis were included in the multivariate analysis, which identified the following independent risk factors for tumour rupture: tumour maximum length ≥5 cm (odds ratio (OR) 1.596; 95% c.i. 1.248 to 2.789; *P* < 0.001), protrusion ratio ≥0.2 (OR 2.784; 95% c.i. 1.746 to 3.943; *P* < 0.001), hepatitis B virus (HBV)-related cirrhosis (OR 1.590; 95% c.i. 1.197 to 2.288; *P* < 0.001), tumour location in the left lobe (OR 1.225; 95% c.i. 1.108 to 1.417; *P* < 0.001), the presence of PVTT (OR 1.342; 95% c.i. 1.157 to 1.790; *P* < 0.001), HBsAg positivity (OR 1.418; 95% c.i. 1.146 to 2.107; *P* = 0.027), hypertension (OR 1.320; 95% c.i. 1.069 to 1.681; *P* = 0.016), and sarcopenia (OR 1.570; 95% c.i. 1.179 to 2.276; *P* = 0.008).

**Table 3:** Univariate and multivariate logistic regression analysis of risk factors related to hepatocellular carcinoma rupture in the training cohort

	Univariate analysis		Multivariate analysis	
	OR*	*P*	OR*	*P*
**Sex**		0.734		
Male	Reference			
Female	1.165 (0.484, 2.801)			
**Age (years)**		0.069		
≤ 60	Reference			
> 60	1.978 (0.948, 4.126)			
**BMI (kg/m^2^)**		0.216		
≤ 30	Reference			
> 30	1.246 (0.891, 1.573)			
**Tumour maximum length (cm)**		<0.001		<0.001
≤ 5	Reference		Reference	
> 5	1.440 (1.146, 2.608)		1.596 (1.248, 2.789)	
**Protrusion from the liver surface**		<0.001		<0.001
No	Reference		Reference	
Yes	1.795 (1.329, 3.015)		1.462 (1.143, 2.796)	
**Protrusion ratio**		<0.001		<0.001
≤ 0.2	Reference		Reference	
> 0.2	2.316 (1.578, 3.814)		2.784 (1.746, 3.943)	
**No. of tumours**		0.155		
Single	Reference			
Multiple	1.146 (0.819, 1.455)			
**Tumour location**		<0.001		<0.001
Left lobe	1.316 (1.187, 1.689)		1.225 (1.108, 1.417)	
Right lobe	Reference		Reference	
Both	1.288 (1.095, 1.572)		1.108 (0.907, 1.334)	
**AFP (ng/ml)**		0.359		
≤ 400	Reference			
> 400	1.346 (0.714, 2.540)			
**Cirrhosis**		0.013		0.008
No	Reference		Reference	
Cause: HBV	1.485 (1.081, 2.718)	<0.001	1.590 (1.197, 2.288)	<0.001
Cause: alcohol	1.246 (0.897, 1.489)	0.135	1.097 (0.762, 1.389)	0.144
Cause: other	1.186 (0.546, 1.590)	0.876	1.080 (0.674, 1.377)	0.744
**CSPH**		0.063		
No	Reference			
Yes	1.208 (0.944, 1.492)			
**Child–Pugh grade**		0.346		
A	Reference			
B	1.147 (0.854, 1.910)			
MELD score (per 1-point increase)	1.021 (0.990, 1.052)	0.146		
**PVTT**		<0.001		<0.001
No	Reference		Reference	
Yes	1.496 (1.276, 1.688)		1.342 (1.157, 1.790)	
**HBsAg**		0.016		0.027
No	Reference		Reference	
Yes	1.331 (1.179, 1.682)		1.418 (1.146, 2.107)	
**HCV**		0.135		
No	Reference			
Yes	1.197 (0.854, 1.435)			
**Drinking history**		0.245		
No	Reference			
Yes	1.346 (0.746, 1.879)			
**Hypertension**		<0.001		0.016
No	Reference		Reference	
Yes	1.204 (1.096, 1.400)		1.320 (1.069, 1.681)	
**Sarcopenia**		<0.001		0.008
No	Reference		Reference	
Yes	1.711 (1.128, 2.578)		1.570 (1.179, 2.276)	
**Albumin (g/L)**		0.349		
≤ 35	Reference			
> 35	1.015 (0.897, 1.230)			
**ALBI score**		0.351		
1	Reference			
2	1.166 (0.619, 1.825)			
3	1.284 (0.846, 1.750)			
**Platelet count (x10³/µL)**		0.119		
≤ 150	Reference			
> 150	0.916 (0.685, 1.416)			
**Creatinine (mg/dl)**		0.287		
≤ 1.2	Reference			
> 1.2	1.490 (0.896, 1.819)			
**Bilirubin (mg/dl)**		0.344		
≤ 1.2	Reference			
> 1.2	1.339 (0.715, 1.914)			

^*^Values in parentheses are 95% confidence intervals. OR, odds ratio; BMI, body mass index; AFP, α-fetoprotein; HBV, hepatitis B virus; CSPH, clinically significant portal hypertension; MELD, Model for End-stage Liver Disease; PVTT, portal vein tumour thrombus; HBsAg, hepatitis B surface antigen; HCV, hepatitis C virus; ALBI, albumin–bilirubin.

### CAPTure, random forest, and deep learning models

**Fig. 2:**
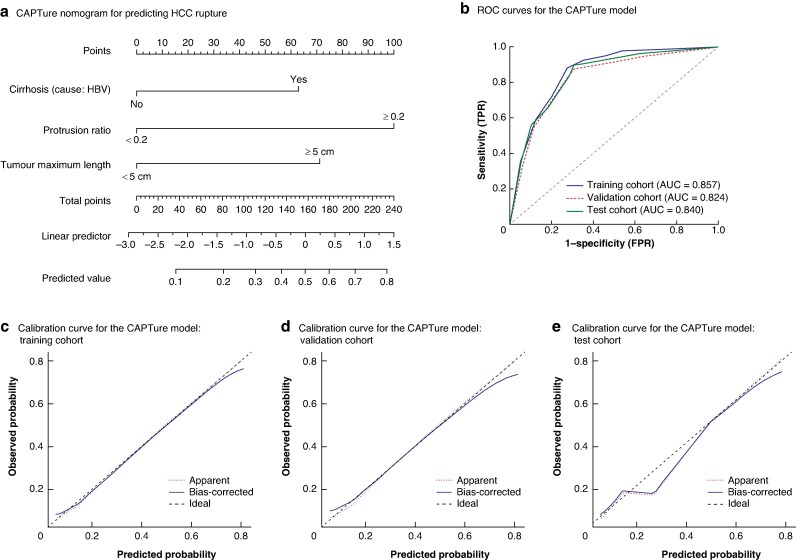
Nomogram and ROC curves for the CAPTure model **a** CAPTure nomogram for predicting HCC rupture based on the three independent predictors, **b** ROC curves for the CAPTure model in the training, validation, and test cohorts, and **c–e** calibration curves for the CAPTure model in the training cohort (**c**), validation cohort (**d**), and test cohort (**e**). ROC, receiver operating characteristic; CAPTure, Cirrhosis, Assessment of Protrusion ratio, and Tumour maximum length; HCC, hepatocellular carcinoma; HBV, hepatitis B virus; TPR, true positive rate; FPR, false positive rate; AUC, area under the curve.

Based on multivariate logistic regression analysis, three independent predictors (cirrhosis assessment, protrusion ratio, and tumour maximum length) were selected to construct the CAPTure nomogram (*Fig. 2*). The area under the ROC curve (AUC) for the nomogram in the training, validation, and test cohorts was 0.857, 0.824, and 0.840, respectively, demonstrating strong discriminatory ability. Calibration curves indicated good consistency between predicted probabilities and observed outcomes across all cohorts (*Fig. 2*).

**Fig. 3:**
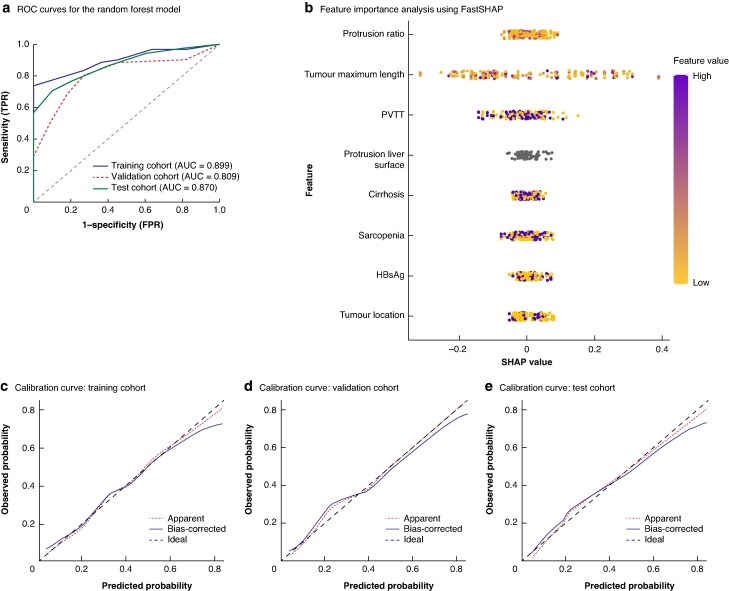
Random forest model **a** ROC curves for the random forest model in the training, validation, and test cohorts, **b** feature importance analysis using FastSHAP, identifying the five most important variables, and **c–e** calibration curves for the training (**c**), validation (**d**), and test (**e**) cohorts. ROC, receiver operating characteristic; FastSHAP, efficient and model-agnostic approximation of SHapley Additive exPlanations; TPR, true positive rate; FPR, false positive rate; AUC, area under the curve; PVTT, portal vein tumour thrombus; HBsAg, hepatitis B surface antigen.

**Fig. 4:**
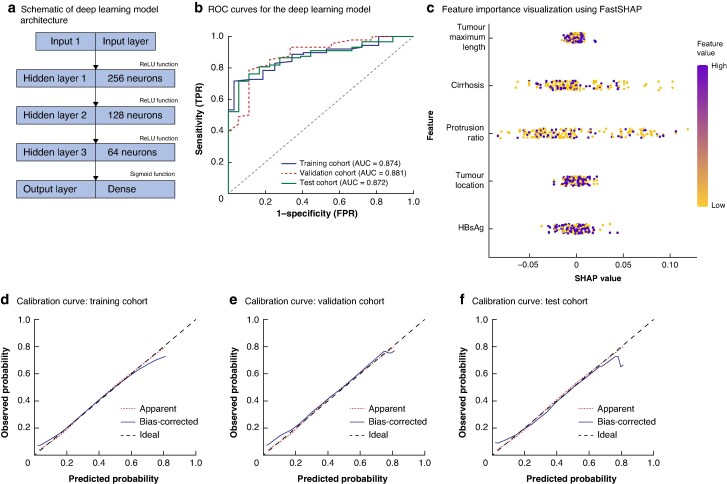
Deep learning model **a** Schematic representation of the deep learning model architecture, comprising three hidden layers with 256, 128, and 64 neurons, ReLU activation for hidden layers, and a sigmoid function in the output layer. **b** ROC curves for the deep learning model across the training, validation, and test cohorts. **c** Feature importance visualization using FastSHAP. **d–f** Calibration curves for the training (**d**), validation (**e**), and test (**f**) cohorts. ReLU, rectified linear unit; FastSHAP, efficient and model-agnostic approximation of SHapley Additive exPlanations; TPR, true positive rate; FPR, false positive rate; AUC, area under the curve; HBsAg, hepatitis B surface antigen.

For the random forest model, parameter optimization resulted in 500 decision trees with a maximum depth of 10. Based on FastSHAP scoring, the model identified tumour maximum length, tumour protrusion beyond the liver surface, protrusion ratio, PVTT, and cirrhosis as the five most important variables. The AUC for the random forest model was 0.899 in the training cohort, 0.809 in the validation cohort, and 0.870 in the test cohort. Calibration curves further confirmed the model’s high predictive accuracy (*Fig. 3*). The deep learning model used three hidden layers with 256, 128, and 64 neurons, using rectified linear unit (ReLU) activation functions for the hidden layers and a sigmoid function for the output layer. The AUC for the deep learning model was 0.874, 0.881, and 0.872 in the training, validation, and test cohorts, respectively. FastSHAP analysis revealed that tumour maximum length, protrusion ratio, and cirrhosis were the most influential predictive factors. Calibration curves demonstrated high consistency between predictions and observations across all data sets (*Fig. 4*).

The CAPTure, random forest, and deep learning models all achieved similar performance metrics across the training, validation, and test cohorts. Detailed results are presented in *Table S4*.

## Discussion

HCC rupture is a severe complication with major prognostic implications. In the present nationwide multicentre cohort, the overall incidence of rupture was 11%, but exceeding 15% in some regions. Once rupture occurs, both short- and long-term outcomes, including survival, deteriorate significantly. This underscores the need for early risk identification and prevention strategies, particularly in Asian populations with higher rupture rates^3–5,12^. To address this issue, rupture-associated risk factors were analysed using a national data set, and the CAPTure model was developed and further enhanced by machine learning algorithms. These tools are intended to facilitate the identification of high-risk patients and support targeted clinical interventions.

Several Asian studies have identified risk factors such as large tumour size, cirrhosis, and hepatitis for HCC rupture. Zhu *et al*.^9^ reported associations of rupture with hypertension, cirrhosis, tumour size > 5 cm, vascular thrombosis, and extrahepatic invasion; Aoki *et al*.^13^ highlighted tumour diameter, Child–Pugh class, des-γ-carboxy prothrombin (DCP) levels, platelet count, and age as factors associated with spontaneous tumour rupture; and Min *et al*.^14^ found advanced Child–Pugh class and peripheral tumour location to be significant factors associated with HCC rupture. In contrast, publications from studies in Western populations are sparse. Obeidat *et al*.^15^ identified obesity and large single tumours as risk factors for tumour rupture, and found cirrhosis to be protective. These apparent discrepancies may stem from the underlying disease aetiologies. In Asia, HBV-related liver disease predominates^16,17^, characterized by chronic inflammation and fibrosis, which promote rapid tumour progression and increase rupture risk^18,19^. HCC in Western patients more often involve alcohol or hepatitis C virus-related disease, with distinct biological pathways. The small room hypothesis suggests that reduced expansibility in cirrhotic livers, especially in the left lobe, increases capsular stress and rupture risk^5,20^. In the present study, the biomechanical effect was quantified using a novel imaging-based metric, namely the protrusion ratio, which reflects the proportion of the tumour extending beyond the liver surface. This parameter was independently associated with rupture risk and was incorporated into the CAPTure model. The strong link between HBV-related cirrhosis and rupture in the present cohorts supports this mechanism. Western studies often focus on general clinical variables, such as body mass index, American Society of Anesthesiologists grade, and albumin level, with limited emphasis on tumour background^15,21^. The findings of the present study align with regional HBV prevalence and emphasize the need to incorporate aetiological context into predictive models. By synthesizing regional differences, this study adds to the understanding of rupture pathogenesis across populations.

A key contribution of this study is the quantification of the protrusion ratio, a previously qualitative feature, and its validation as an independent predictor of rupture risk. The CAPTure model, incorporating cirrhosis, protrusion ratio, and tumour size, offers a practical and clinically applicable tool for risk prediction. Previous models, such as those developed by Wu *et al*.^22^ and Ye *et al*.^23^, included factors like ascites, cirrhosis, and tumour protrusion but the studies were limited by a single-centre design, small cohorts, or complex scoring systems. By transforming subjective assessments into quantifiable metrics, the CAPTure model enables rapid estimation of rupture risk, supporting clinical decision-making. In the present study, the CAPTure model demonstrated good discriminatory performance across training, validation, and test cohorts. To complement it, machine learning models (random forest and deep learning) were developed, which showed comparable accuracy and captured non-linear relationships between predictors. Integration of FastSHAP^11^ allowed interpretable ranking of feature importance, with tumour size, protrusion ratio, and cirrhosis consistently emerging as the top predictors, supporting the CAPTure variable selection. Although all models showed similar predictive power, the machine learning approaches provide added flexibility in handling complex variable interactions, which may improve patient stratification. For individuals identified as being at high risk of tumour rupture, intensified imaging surveillance and early intervention (for example TACE) should be considered^24–26^. Furthermore, optimizing antiviral and antifibrotic therapies may lower rupture risk in patients with cirrhosis, improving overall outcomes.

Beyond HBV-related cirrhosis and the protrusion ratio, several independent predictors identified in the multivariable analysis warrant further consideration. Larger (> 5 cm) tumours may increase intratumoural pressure and capsular tension, predisposing to rupture. PVTT contributes to vascular congestion and elevated intratumoural pressure, increasing haemorrhagic risk. Tumours in the left hepatic lobe may be more vulnerable because of the smaller volume and reduced expansibility of the left lobe, particularly in cirrhotic livers with diminished compliance. Sarcopenia, reflecting systemic inflammation and poor nutritional status, may impair tissue resilience and healing capacity, weakening structural integrity. Hypertension may exacerbate microvascular stress, further destabilizing the tumour environment. These observations support a multifactorial pathophysiological model for spontaneous HCC rupture, underscoring the need to consider clinical, anatomical, and systemic factors in comprehensive risk assessment.

For high-risk patients identified by the models described in this study, personalized strategies, such as close imaging surveillance, TACE, or surgery, should be considered. Optimizing antiviral and antifibrotic therapies may also reduce rupture risk in cirrhotic patients, thereby improving outcomes. Beyond identifying high-risk individuals, the CAPTure and machine learning models can guide clinical decision-making and resource prioritization, especially in resource-limited settings common in Asia^27^. These tools may also support dynamic follow up during post-treatment monitoring, enabling earlier detection of rupture risk and timely intervention. The CAPTure nomogram complements traditional staging systems like the Barcelona Clinic Liver Cancer (BCLC) strategy by providing an objective, imaging-based tool to stratify patients according to rupture risk. This facilitates timely intervention, such as early resection, neoadjuvant locoregional therapy, or intensified surveillance, especially in patients with exophytic tumours or PVTT. In complex cases, such as patients with BCLC stage C and high-risk features, the nomogram can aid multidisciplinary discussions and inform treatment modifications that reduce rupture risk before initiating systemic therapy. Importantly, the model relies on variables routinely available in clinical practice, supporting its utility in HBV-endemic and resource-constrained environments. Thus, the CAPTure model bridges the gap between predictive analytics and real-world clinical application, enabling proactive, individualized management for patients at risk of spontaneous HCC rupture.

The strengths of the present study include a large national sample size, multicentre collaboration, and rigorous methodology, which collectively provide robust evidence for the conclusions drawn. By applying advanced statistical techniques and incorporating both traditional and machine learning models, this study offers a comprehensive framework for understanding rupture risk in HCC. However, several limitations must be acknowledged. First, the retrospective design inherently introduces potential selection bias and limits the ability to infer causality. Second, the study population was drawn from specialized tertiary centres, which may not fully represent the general HCC patient population, thereby affecting the generalizability of the findings. Third, although the results of the present study support the space constraint hypothesis, further mechanistic and prospective studies are required to validate this concept and clarify the underlying biological mechanisms.

This study analysed key risk factors for HCC rupture in an Asian population and developed predictive models, including the CAPTure model and two machine learning algorithms, to support early risk identification. The association between HBV-related cirrhosis and rupture highlights the need for region-specific prevention strategies and more efficient resource allocation. Future work should focus on prospective multicentre validation and explore the integration of imaging features, inflammatory markers, and liver stiffness into prediction models. These tools may be embedded into clinical workflows to guide surveillance and treatment in high-risk patients. The findings of the present study lay a foundation for targeted interventions and improved management of this serious complication.

## Supplementary Material

zraf105_Supplementary_Data

## Data Availability

The data sets generated and/or analysed during the present study are not publicly available due to institutional and ethical restrictions. However, they are available from the corresponding author upon reasonable request and with appropriate justification.
