# Supplementary material for: Risk factors for hepatocellular carcinoma rupture: multicentre retrospective study
Source: BJS Open. 2025 Nov 4;9(6):zraf105. doi: 10.1093/bjsopen/zraf105 (PMC12586324; doi:10.1093/bjsopen/zraf105)
Supplement: zraf105_Supplementary_Data [file zraf105_supplementary_data.docx]

**Title:** Risk Factors for Hepatocellular Carcinoma Rupture: Multicenter Retrospective Study

Feng Xia^1*a^, Yiyang Liu^1.2*a^, Hongwei Huang^1*a^, Xulin Liu^1^, Jing Yan^3^, Zhancheng Qiu^4^, Qiao Zhang^5^, Zhenheng Wu^6^, Zhiyuan Huang^7^, Renjie Wei^8^, Li Lin^9^, Liping Liu^10^, Shuangqin Han^11^, Yulin Yuan^12^, Huaxuan Yin^13^, Guobing Xia^14^, Yunyan Wan^15^, Shuo Xiao^16^, Guoxiang Zhou^17^, Xiafei Xia^18^, Huapeng Sun^19^, Shuai Wang^20^, Jun Zheng^21^, Hengyi Gao^22^, Jiang Zheng^23^, Li Ren^24^, Ali Mo^25^, Lin Ye^26^, Shun Ruan^27^, Xiaoping Chen^1*b^, Qi Cheng^1*b^, Bixiang Zhang^1*b^, Peng Zhu^1*b^

1. Department of Hepatic Surgery, Tongji Hospital, Tongji Medical College of Huazhong University of Science and Technology, Wuhan, Hubei, China
2. Department of Hepatobiliary Surgery, Union Hospital, Tongji Medical College, Huazhong University of Science and Technology, Wuhan, Hubei, China
3. Department of Ultrasound in Medicine, The Second Affiliated Hospital of Zhejiang University School of Medicine, Zhejiang, China.
4. Department of General Surgery, West China Hospital, Sichuan University, Chengdu, Sichuan Province, China.
5. Department of Hepatic Surgery, Zhongshan People's Hospital Affiliated to Guangdong Medical University, Zhongshan, Guangdong, China
6. Department of Hepatopancreatobiliary Surgery, The First Affiliated Hospital of Fujian Medical University, Fuzhou, Fujian, 350001, China.
7. Department of General Surgery, General Hospital of Central Theater Command, Wuhan, Hubei, China.
8. Department of Neurosurgery, Zhongnan Hospital of Wuhan University, Wuhan, Hubei, China.
9. Clinic Center of Human Gene Research, Union Hospital, Tongji Medical College, Huazhong University of Science and Technology, Wuhan, Hubei, China.
10. Department of Hepatobiliary Surgery, Shenzhen People's Hospital, Shenzhen, Guangdong, China.
11. Department of Hepatobiliary Surgery, Tianjin First Central Hospital, Tianjin Medical University, Tianjin , China
12. School of Medicine, Wuhan University of Science and Technology, Wuhan, Hubei, China
13. Department of Hepatic-biliary-pancreatic Surgery, The First People's Hospital of Foshan, Foshan ,China
14. Department of Hepatobiliary and Pancreatic Surgery, Huangshi Central Hospital, Hubei Polytechnic University .Huangshi, Hubei, China.
15. Department of Hepatobiliary Pancreatic Surgery, Taihe Hospital, Shiyan City, Hubei Province, China.
16. Department of Cardiac Surgery, Guangdong Provincial People’s Hospital, Southern Medical University, Guangzhou, Guangdong, China
17. Department of Heart Center, Women and Children's Hospital of Qingdao University, Qingdao, Shandong, China.
18. Department of Organ Transplantation, The First Affiliated Hospital of Kunming Medical University, Kunming Medical University, Kunming, China.
19. Department of General Surgery, Xiangyang Central Hospital, Affiliated Hospital of Hubei University of Arts and Science, Xiangyang, China.
20. Department of Hepatobiliary Surgery, Jingzhou Central Hospital, Jingzhou, China.
21. Department of Science and Education, Shenzhen Baoan District People's Hospital, Guangdong, China.
22. Department of Hepatobiliary and Pancreatic Surgery, Shenzhen Longhua District People's Hospital, Guangdong, China.
23. Department of Cardiology, Wuhan Yaxin General Hospital Affiliated to Wuhan University of Science and Technology, Wuhan, Hubei, China
24. Department of Hepatobiliary Surgery, Affiliated Hospital of Qinghai University, Xining, Qinghai, China.
25. Department of General Surgery, First Affiliated Hospital of Shaoyang University, Shaoyang City, Hunan, China.
26. Department of Hepatobiliary and Pancreatic Surgery, Affiliated Hospital of Guilin Medical University, Guilin, Guangxi Zhuang Autonomous Region, China
27. Department of Surgery, Dongguan Hepatobiliary Hospital, Dongguan, Guangdong, China

a.These authors contributed equally to the article and should be listed as co-first authors.

b. These authors are listed as co-corresponding authors.

**Corresponding authors:**

Peng Zhu, MD, Department of Hepatic Surgery, Tongji Hospital, Tongji Medical College of Huazhong University of Science and Technology, 1095, Jiefang Avenue, Wuhan, China. (zhupeng@tjh.tjmu.edu.cn).

Bixiang Zhang, MD, Department of Hepatic Surgery, Tongji Hospital, Tongji Medical College of Huazhong University of Science and Technology, 1095, Jiefang Avenue, Wuhan, China. (bixiangzhang@163.com).

Qi Cheng, MD, Department of Hepatic Surgery, Tongji Hospital, Tongji Medical College of Huazhong University of Science and Technology, 1095, Jiefang Avenue, Wuhan, China. (chengqi@hust.edu.cn).

Xiaoping Chen, MD, Department of Hepatic Surgery, Tongji Hospital, Tongji Medical College of Huazhong University of Science and Technology, 1095, Jiefang Avenue, Wuhan, China. (chenxpchenxp@163.com).

**Supplementary Materials - Index**

| Supplementary Methods | pag. 6-11 |
| --- | --- |
| Supplementary Figures and Tables |  |
| Supplementary Figure 1 | pag. 12 |
| Supplementary Figure 2 | pag. 13 |
| Supplementary Figure 3 | pag. 14 |
| Supplementary Figure 4 | pag. 15 |
| Supplementary Table 1 | pag. 16-18 |
| Supplementary Table 2 | pag. 19-21 |
| Supplementary Table 3 | pag. 22 |
| Supplementary Table 4 | pag. 23 |

Supplementary Figure Legends

**Supplementary Figure 1.**

**Kaplan-Meier Curve for Overall Survival (OS) in Ruptured and Non-Ruptured HCC Patients.**
The Kaplan-Meier survival curve illustrates a significant difference in OS between the rupture and non-rupture groups. The hazard ratio for OS in ruptured patients was 2.45 (95% CI, 2.22–2.69; P < 0.001). Survival rates were markedly lower in the ruptured group across the 60-month follow-up period.

**Supplementary Figure 2.**

**Kaplan–Meier Curve for Overall Survival (OS) After PSM.**

The Kaplan–Meier survival curve shows a significant difference in OS between ruptured and non-ruptured HCC patients after propensity score matching. The hazard ratio for OS in the rupture group was 2.36 (95% CI, 2.06–2.72; P < 0.001).

**Supplementary Figure 3.**

**Kaplan–Meier Curve for Overall Survival (OS) in Ruptured and Non-Ruptured HCC Patients Undergoing Hepatectomy.**

This Kaplan–Meier curve illustrates the difference in OS among patients who underwent liver resection. The hazard ratio for OS in the rupture group was 2.28 (95% CI, 1.99–2.62; P < 0.001).

**Supplementary Figure 4.**

**Youden Index Analysis for Determining the Optimal Cutoff for Protrusion Ratio.**
The ROC curve highlights the optimal cutoff value for the Protrusion Ratio, determined to be 0.2051 (AUC = 0.800, 95% CI, 0.760–0.840). This cutoff value was set at 0.2, providing strong discriminatory power for predicting rupture risk. ​

Supplementary Methods

**Definitions**

HCC diagnosis based on imaging and serum markers required: (1) the presence of characteristic arterial phase hyperenhancement with washout in the portal or delayed phase on contrast-enhanced imaging (Computed Tomography (CT) or Magnetic Resonance Imaging (MRI)); and (2) elevated serum alpha-fetoprotein (AFP) level >400 ng/mL. Diagnosis adhered to the diagnostic criteria outlined by the American Association for the Study of Liver Diseases (AASLD) and the European Association for the Study of the Liver (EASL) Clinical Practice Guidelines. Although some patients also underwent abdominal ultrasound or magnetic resonance imaging (MRI) during their diagnostic workup, these modalities were not used to determine rupture status due to their lower temporal resolution and limited sensitivity for detecting peritumoral bleeding. Consequently, only contrast-enhanced CT findings were used for radiological confirmation of ruptured hepatocellular carcinoma (rHCC). Sarcopenia was diagnosed using skeletal muscle index (SMI) calculated from imaging data (CT or MRI) at the third lumbar vertebra level. Cutoff values were set at <50 cm²/m² for men and <39 cm²/m² for women, based on established criteria; Hypertension was defined as a systolic blood pressure ≥140 mmHg or diastolic blood pressure ≥90 mmHg on at least two separate occasions, or the documented use of antihypertensive medications; Clinically Significant Portal Hypertension (CSPH) was defined as a hepatic venous pressure gradient (HVPG) ≥10 mmHg or the presence of indirect indicators, including splenomegaly with thrombocytopenia (platelet count <100 × 10⁹/L), esophageal or gastric varices, or radiological evidence of portosystemic collaterals. AFP levels were measured at baseline, and a standardized cutoff value of 400 ng/mL was applied, consistent with prior literature and international practice guidelines. This value was used for stratification in baseline characteristics and regression modeling but not as a diagnostic threshold. A tumor diameter cutoff of 5 cm was adopted based on its widespread use in prior studies analyzing predictors of HCC rupture. This threshold reflects a clinically relevant size at which the risk of rupture is believed to increase due to higher intratumoral pressure and capsular stress.

**Diagnostic Adjudication in Cases of Discordant Imaging and AFP Results**

In situations where imaging findings were suggestive of HCC but serum AFP levels were ≤400 ng/mL (or vice versa), final classification was based on one of the following:

(1) histopathological confirmation from surgical specimens or biopsies when available;

(2) if pathology was not available, classification was made by consensus among two experienced radiologists and one hepatobiliary surgeon after comprehensive review of imaging features and clinical context. This adjudication process ensured consistency and minimized diagnostic ambiguity in borderline cases.

**Cirrhosis Etiology Definition**

The classification of cirrhosis etiology was determined through a comprehensive clinical assessment, rather than solely on viral marker positivity or alcohol history.

HBV-related cirrhosis was defined as the presence of HBsAg positivity along with radiological signs of chronic liver disease (e.g., nodular liver surface, splenomegaly, or collateral circulation) and/or documented physician diagnosis in the patient’s medical records.

Alcohol-related cirrhosis was defined by a chronic alcohol consumption history in combination with imaging features consistent with cirrhosis or a formal diagnosis recorded by the managing clinician.

Patients with viral infection or alcohol use but without supportive clinical or imaging evidence of cirrhosis were not classified as cirrhotic.

**Development and Validation of the Nomogram**

The nomogram was constructed based on the results of multivariate logistic regression analysis. Initially, all candidate variables were evaluated for their association with HCC rupture. Three variables—cirrhosis status, protrusion ratio, and tumor maximum length—demonstrated the strongest statistical significance (P < 0.05) and the highest odds ratios (OR), indicating their roles as independent risk factors.

Although multiple independent predictors of rupture were identified through multivariate logistic regression—including PVTT, hypertension, HBsAg positivity, and sarcopenia—the final CAPTure nomogram was constructed using only three key variables: cirrhosis (yes/no), tumor diameter >5 cm, and protrusion ratio ≥0.2.

These variables were selected based on their:

(1) consistently strong statistical significance and contribution across datasets,

(2) low multicollinearity with other features,

(3) clinical interpretability and routine availability without need for advanced processing, and

(4) endorsement from multidisciplinary clinical experts as practical and actionable.

This balance ensured that the model would be both predictive and easily applicable in varied real-world clinical settings. Using these variables, the nomogram was developed to estimate the probability of tumor rupture. The regression coefficients of the selected variables were used to assign proportional scores within the nomogram, ensuring accurate contributions to the overall risk prediction.

To validate the nomogram, calibration curves were generated to assess the consistency between predicted and observed probabilities. The discriminative ability of the nomogram was evaluated using receiver operating characteristic (ROC) curves, with the area under the curve (AUC-ROC) calculated as a measure of predictive accuracy. Key performance metrics, including accuracy, sensitivity, and specificity, further supported the nomogram’s reliability as a practical tool for clinical application.

**Machine Learning Models: Random Forest and Deep Learning**

**Random Forest**

To further enhance predictive performance, we also employed random forest and deep learning models, leveraging their ability to capture nonlinear relationships and interpret variable importance. The random forest model, an ensemble learning method, was employed to improve predictive accuracy and handle the complexity of high-dimensional data. The model consisted of 500 decision trees, each trained on a random subset of data and features. The final prediction was obtained by averaging the outputs of all trees. To balance model complexity and prevent overfitting, the maximum depth of each tree was limited to 10. Hyperparameters, including the number of trees and the minimum samples required to split a node, were optimized using grid search on the validation set.

**Deep Learning**

A fully connected neural network was developed to capture complex nonlinear relationships among variables. The architecture included three hidden layers, with 256, 128, and 64 neurons, respectively. ReLU activation functions were applied to all layers except for the final layer, which used a sigmoid activation function to output probabilities. Hyperparameters, including the number of neurons in each layer and the learning rate, were fine-tuned using the validation set to maximize performance.

**Model Evaluation**

Due to variations in patient numbers across centers, the dataset was divided into training (70%), validation (15%), and test (15%) sets based on overall proportions. This approach ensured that the training set contained sufficient data to develop optimal predictive models, while the validation and test sets remained representative. Including data from all centers in each subset allowed the model to comprehensively learn population-wide characteristics, improving robustness and generalizability.

Training Cohort: Used for model development, forming the core data for building random forest and deep learning models.

Validation Cohort: Used for hyperparameter tuning and intermediate performance evaluation. For random forest, parameters such as maximum depth and the number of trees were optimized; for deep learning, learning rates and neuron numbers were adjusted based on validation performance.

Test Cohort: Independently assessed the final model's generalization capability, ensuring unbiased evaluation.

To further enhance robustness, 10-fold cross-validation was performed on the training set. The training data were randomly split into 10 subsets, with 9 subsets used for training and 1 for validation in each iteration. This process was repeated 10 times, and performance metrics (e.g., AUC-ROC, accuracy, recall) were averaged to provide reliable results. The optimal model configuration determined during hyperparameter tuning was applied to the test set for final evaluation.

**Performance Metrics**

Key performance metrics for the traditional model, random forest, and deep learning models included AUC-ROC, sensitivity, specificity, and calibration. Both machine learning models achieved strong predictive accuracy and effectively captured complex nonlinear interactions.

**FastSHAP Integration**

Fast SHapley Additive exPlanations (SHAP) was used to visualize variable importance in both models. This method efficiently computes Shapley values to explain each variable's contribution to the predictive outcome. Compared to traditional SHAP, FastSHAP improves computational efficiency while maintaining accuracy and consistency. It assessed both global feature contributions across all samples and local contributions for individual cases. Using FastSHAP, we identified the influence of specific variables, such as protrusion ratio, on rupture risk predictions, offering intuitive insights into model decision-making. These machine learning models complemented the performance of the nomogram and provided alternative methods for precise risk assessment.

## Follow-up

## Patients were followed up every 3 months post-discharge. Follow-up evaluations included imaging examinations (e.g., contrast-enhanced CT and abdominal MRI) and laboratory tests (e.g., liver and kidney function, electrolytes, and tumor markers). OS was defined as the time from the first day of diagnosis upon admission to the date of death. Follow-up continued until June 30, 2024.

**Propensity Score Matching (PSM)**
To reduce selection bias and balance baseline characteristics between the rupture and non-rupture groups, a 1:1 propensity score matching (PSM) analysis was performed using the nearest-neighbor method without replacement. The propensity score was estimated using a multivariable logistic regression model that included the following covariates: tumor diameter, PVTT, cirrhosis status, HBsAg positivity, sarcopenia, and hypertension. A caliper width of 0.1 was applied to ensure adequate matching quality. After matching, survival analyses were repeated within the matched cohort to evaluate the independent prognostic impact of tumor rupture.

Supplementary Figures and Tables

Supplementary Figure 1


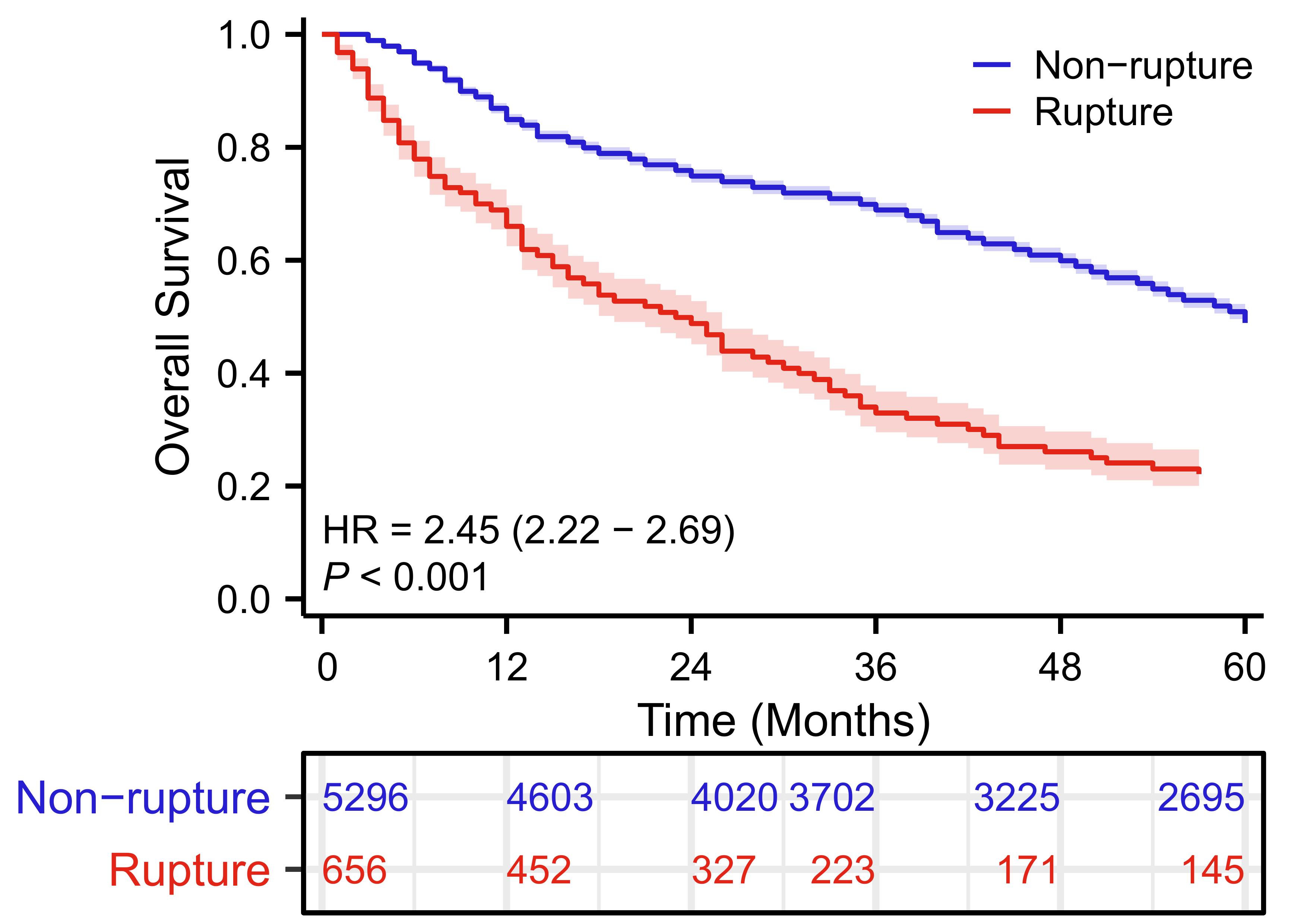


Supplementary Figure 2


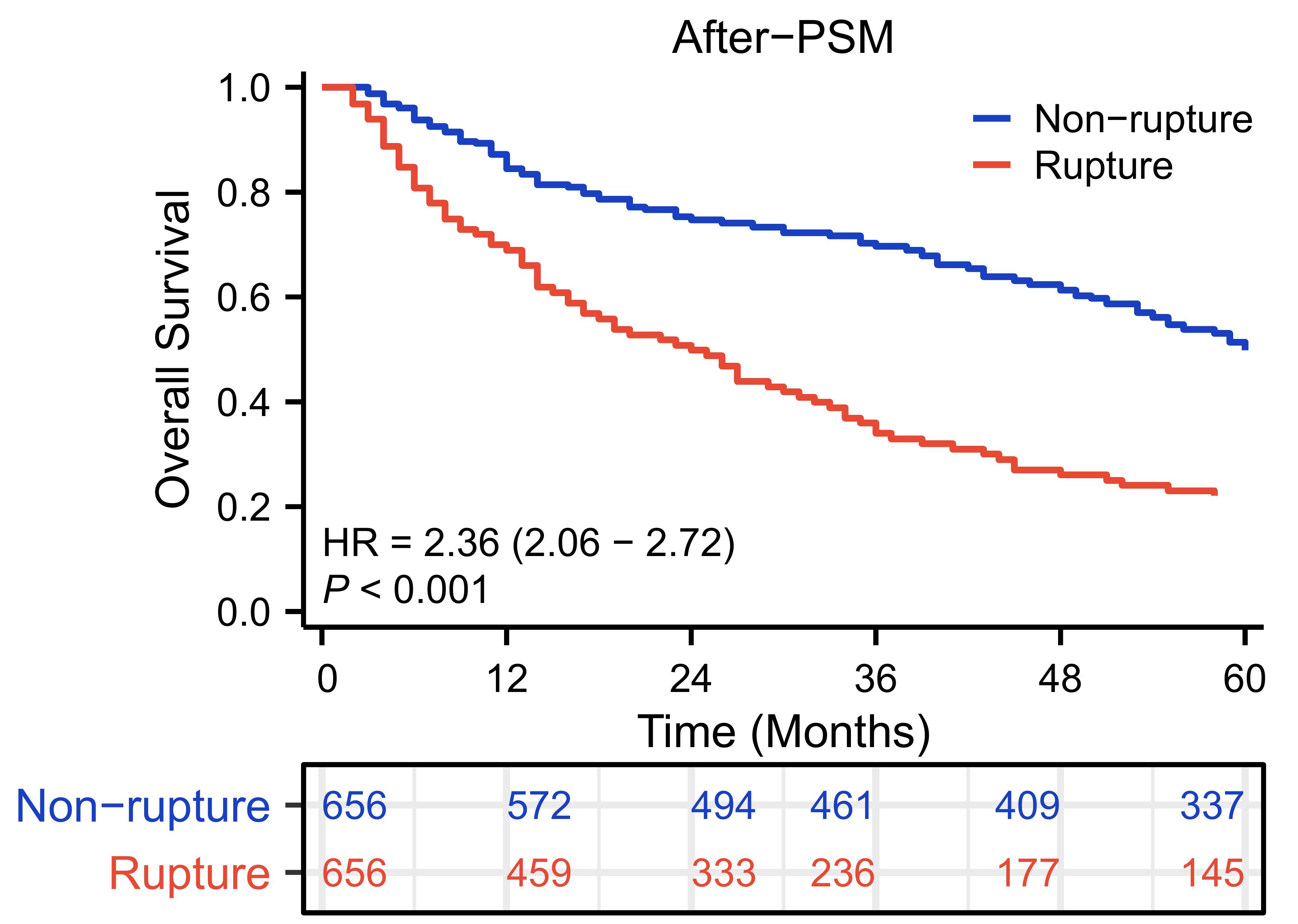


Supplementary Figure 3


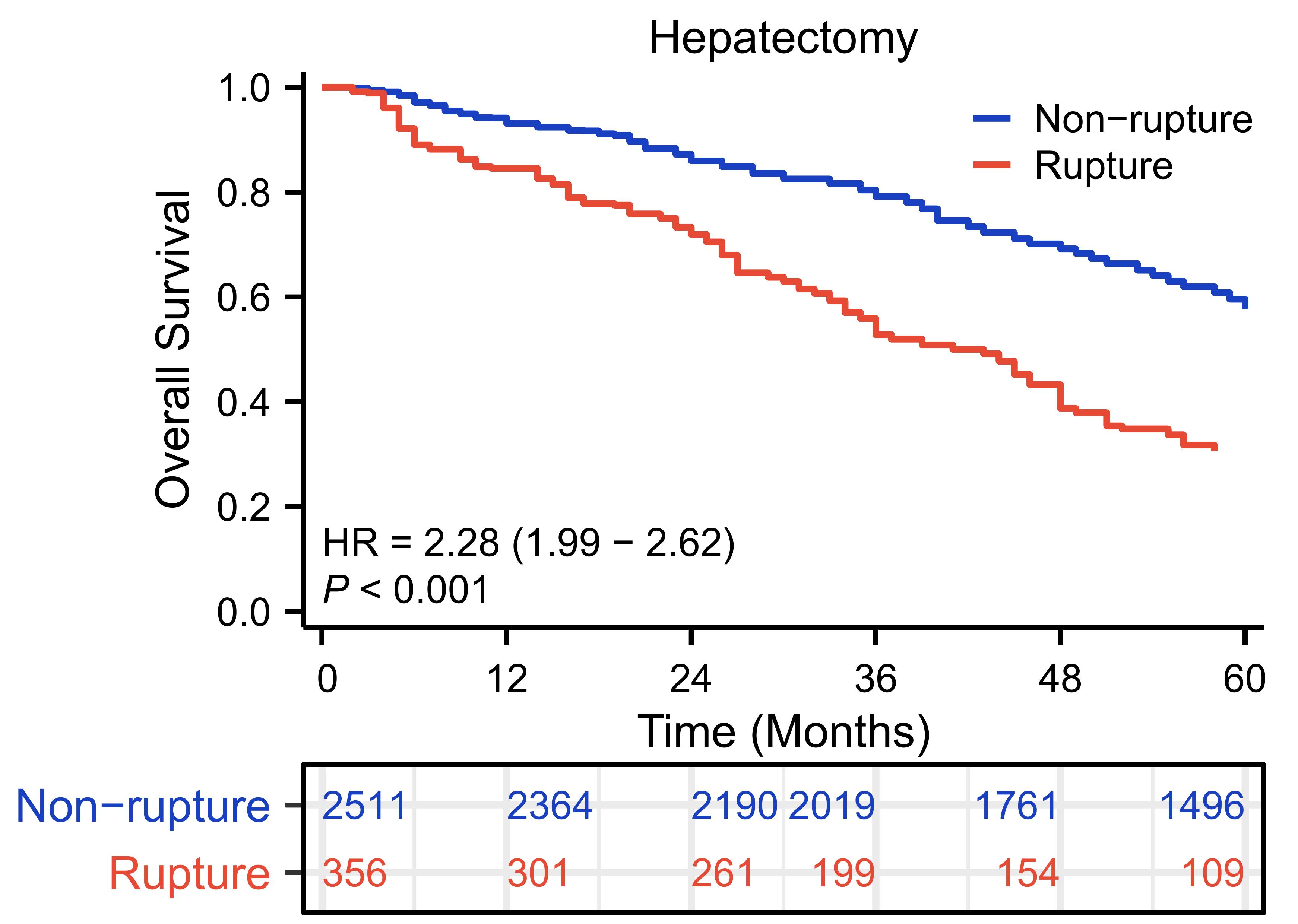


Supplementary Figure 4


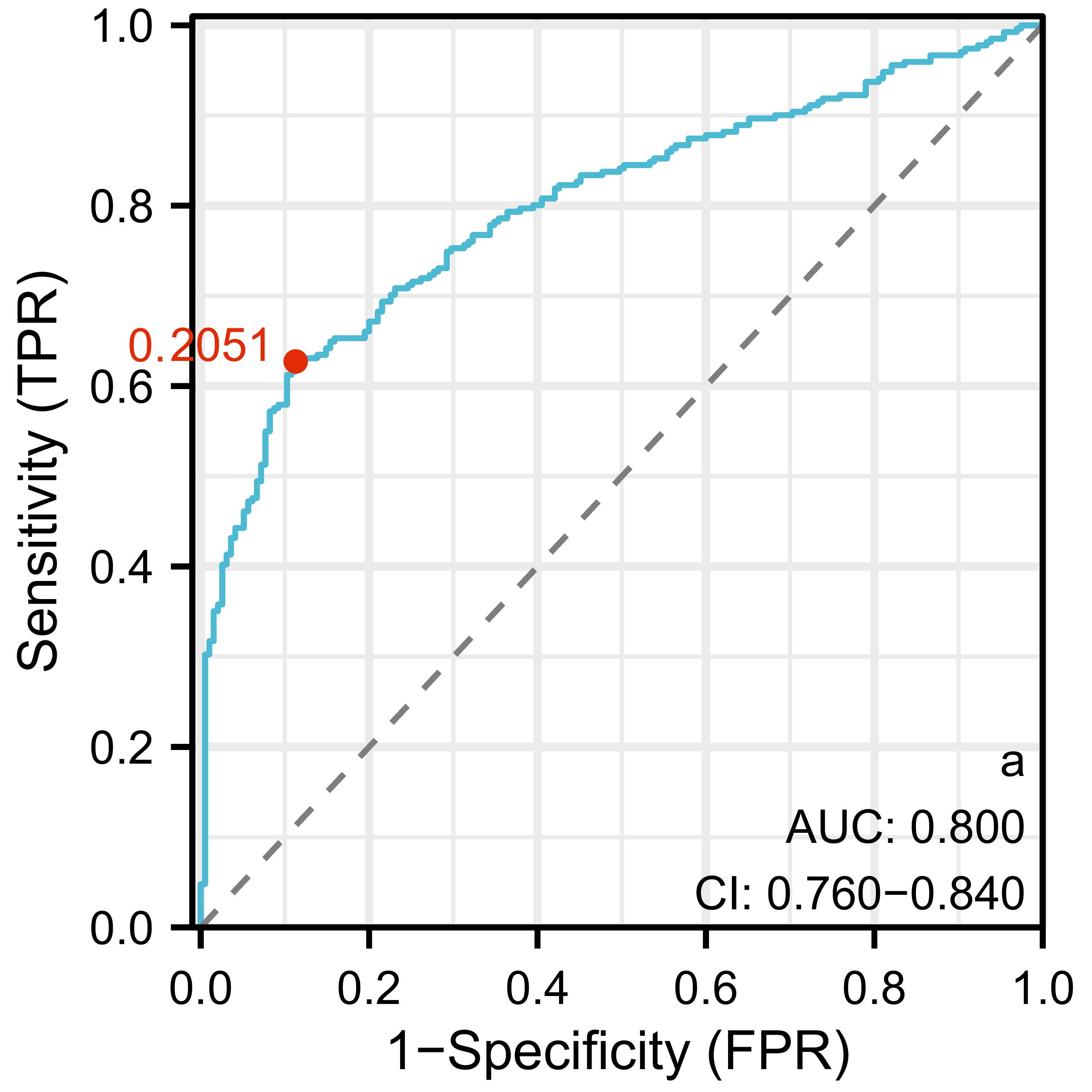


**Supplementary Table 1. Baseline characteristics of patients with HCC in the rupture and Non-rupture cohorts after PSM.**

|  |  | **Rupture (n=656)** | **Non-rupture (n=656)** | **P-Value‡** |
| --- | --- | --- | --- | --- |
| Gender (%) |  |  |  | 0.110 |
|  | Male | 558 (85.1) | 569 (88.1) |  |
|  | Female | 98 (14.9) | 77 (11.9) |  |
| Age (%) |  |  |  | 0.845 |
|  | ≤ 60 y | 500 (76.2) | 503 (76.7) |  |
|  | > 60 y | 156 (23.8) | 153 (23.3) |  |
| BMI (%) |  |  |  | 0.298 |
|  | ≤ 30kg/m^2^ | 611 (93.1) | 601 (91.6) |  |
|  | > 30kg/m^2^ | 45 (6.9) | 55 (8.4) |  |
| Tumor max length (%) |  |  |  | 0.628 |
|  | ≤ 5 cm | 198 (30.2) | 190 (29.0) |  |
|  | > 5 cm | 458 (69.8) | 466 (71.0) |  |
| Protrusion from the liver surface (%) |  |  |  | 0.233 |
|  | No | 162 (24.7) | 181 (27.6) |  |
|  | Yes | 494 (75.3) | 475 (72.4) |  |
| Tumor number (%) |  |  |  | 0.540 |
|  | Single | 494 (75.3) | 505 (76.7) |  |
|  | Multiple | 162 (24.7) | 153 (23.3) |  |
| Tumor location (%) |  |  |  | 0.877 |
|  | Left | 275 (41.9) | 284 (43.3) |  |
|  | Right | 301 (45.9) | 295 (45.0) |  |
|  | Both | 80 (12.2) | 77 (11.7) |  |
| AFP (%) |  |  |  | 0.508 |
|  | ≤ 400ng/ml | 316 (48.2) | 328 (50.0) |  |
|  | > 400ng/ml | 340 (51.8) | 328 (50.0) |  |
| Cirrhosis (%) |  |  |  | 0.408 |
|  | No | 100 (15.2) | 111 (16.9) |  |
|  | Yes | 556 (84.8) | 545 (83.1) |  |
| CSPH (%) |  |  |  | 0.353 |
|  | No | 484 (73.8) | 469 (71.5) |  |
|  | Yes | 172 (26.2) | 187 (28.5) |  |
| Child-Pugh (%) |  |  |  | 0.652 |
|  | A | 396 (60.4) | 388 (59.1) |  |
|  | B | 260 (39.6) | 268 (40.9) |  |
| MELD |  | 10.1 (8.4–13.0) | 9.6 (8.0–12.4) | 0.203 |
| PVTT (%) |  |  |  | 0.590 |
|  | Presence | 196 (29.9) | 205 (31.3) |  |
|  | Absence | 460 (70.1) | 451 (68.8) |  |
| HBsAg (%) |  |  |  | 0.693 |
|  | No | 54 (8.2) | 58 (8.8) |  |
|  | Yes | 602 (91.8) | 598 (91.2) |  |
| HCV* (%) |  |  |  | 1.000 |
|  | No | 654 (99.7) | 654 (99.7) |  |
|  | Yes | 2 (0.3) | 2 (0.3) |  |
| Drink history (%) |  |  |  | 0.677 |
|  | No | 454 (69.2) | 447 (68.1) |  |
|  | Yes | 202 (30.8) | 209 (31.9) |  |
| Hypertension (%) |  |  |  | 0.584 |
|  | No | 471 (71.8) | 462 (70.4) |  |
|  | Yes | 185 (28.2) | 194 (29.6) |  |
| Sarcopenia (%) |  |  |  | 0.738 |
|  | No | 282 (43.0) | 288 (43.9) |  |
|  | Yes | 374 (57.0) | 368 (56.1) |  |
| ALB (%) |  |  |  | 0.617 |
|  | ≤ 35g/L | 298 (45.4) | 289 (44.1) |  |
|  | > 35g/L | 358 (54.6) | 367 (55.9) |  |
| ALT (%) |  |  |  | 0.304 |
|  | ≤ 50U/L | 530 (80.8) | 515 (78.5) |  |
|  | > 50U/L | 126 (19.2) | 141 (21.5) |  |
| AST (%) |  |  |  | 0.371 |
|  | ≤ 40U/L | 372 (56.7) | 388 (59.1) |  |
|  | > 40U/L | 284 (43.3) | 268 (40.9) |  |
| ALP (%) |  |  |  | 0.688 |
|  | ≤ 100U/L | 510 (77.7) | 516 (78.7) |  |
|  | > 100U/L | 146 (22.3) | 140 (21.3) |  |
| GGT (%) |  |  |  | 0.616 |
|  | ≤ 60U/L | 378 (57.6) | 369 (56.3) |  |
|  | > 60U/L | 278 (42.4) | 287 (43.8) |  |
| ALBI grade (%) |  |  |  | 0.574 |
|  | 1 | 596 (90.9) | 588 (89.4) |  |
|  | 2 | 51 (7.8) | 57 (8.7) |  |
|  | 3 | 9 (1.4) | 13 (2.0) |  |
| Platelets (%) |  |  |  | 0.632 |
|  | ≤ 150/mm^3^ | 138 (21.0) | 131 (20.0) |  |
|  | > 150/mm^3^ | 518 (79.0) | 525 (80.0) |  |
| Creatinine (%) |  |  |  | 0.807 |
|  | ≤ 1.2 mg/dL | 648 (98.8) | 647 (98.6) |  |
|  | > 1.2 mg/dL | 8 (1.2) | 9 (1.4) |  |
| Bilirubin (%) |  |  |  | 0.597 |
|  | ≤ 1.2 mg/dL | 641 (97.7) | 638 (97.3) |  |
|  | > 1.2 mg/dL | 15 (2.3) | 18 (2.7) |  |
| Albumin (%) |  |  |  | 0.321 |
|  | ≤ 35 g/L | 108 (16.5) | 95 (14.5) |  |
|  | > 35 g/L | 548 (83.5) | 561 (85.5) |  |

The values in parentheses are percentages unless indicated otherwise.

^‡^ χ^2^ test with Yates’ correction.

*Indicates the use of Fisher's exact test.

Abbreviations: AFP: alpha-fetoprotein; ALP: alkaline phosphatase; ALT: alanine aminotransferase; AST: aspartate aminotransferase; BMI: Body Mass Index; CSPH: Clinically Significant Portal Hypertension; MELD: Model for End-Stage Liver Disease; GGT: γ-glutamyl transpeptidase; HBsAg: hepatitis B surface antigen; HCC: hepatocellular carcinoma; HCV: Hepatitis C Virus; PVTT: Portal Vein Tumor Thrombus

**Supplementary Table 2. Baseline characteristics of HCC patients with/without ruptured in the training, validation and test cohorts (n = 5952).**

|  |  | **Training cohort** | **Validation cohort** | **Test cohort** | **P-Value‡** |
| --- | --- | --- | --- | --- | --- |
| N |  | 4166 | 892 | 894 |  |
| Gender (%) |  |  |  |  | 0.505 |
|  | Male | 3518 (84.4) | 749 (84.0) | 741 (82.9) |  |
|  | Female | 648 (15.6) | 143 (16.0) | 153 (17.1) |  |
| Age (%) |  |  |  |  | 0.281 |
|  | ≤60 y | 3160 (75.9) | 698 (78.3) | 688 (77.0) |  |
|  | >60 y | 1006 (24.1) | 194 (21.7) | 206 (23.0) |  |
| BMI (%) |  |  |  |  | 0.936 |
|  | ≤30kg/m^2^ | 3867 (92.8) | 831 (93.2) | 8319 (93.0) |  |
|  | >30kg/m^2^ | 299 (7.2) | 61 (6.8) | 63 (7.0) |  |
| Tumor max length (%) |  |  |  |  | 0.620 |
|  | ≤5 cm | 2004 (48.1) | 434 (48.7) | 446 (49.9) |  |
|  | >5 cm | 2162 (51.9) | 458 (51.3) | 448 (50.1) |  |
| Protrusion from the liver surface (%) |  |  |  |  | 0.983 |
|  | No | 2172 (52.1) | 462 (51.8) | 466 (52.1) |  |
|  | Yes | 1994 (47.9) | 430 (48.2) | 428 (47.9) |  |
|  | Protrusion ratio | 0.14±0.06 | 0.14±0.05 | 0.14±0.06 | 1.000 |
| Tumor number (%) |  |  |  |  | 0.869 |
|  | Single | 3162 (75.9) | 678 (76.0) | 686 (76.7) |  |
|  | Multiple | 1004 (24.1) | 214 (24.0) | 208 (23.3) |  |
| Tumor location (%) |  |  |  |  | 0.762 |
|  | Left | 1067 (25.6) | 228 (25.6) | 229 (25.6) |  |
|  | Right | 2548 (61.2) | 556 (62.3) | 559 (62.5) |  |
|  | Both | 551 (13.2) | 108 (12.1) | 106 (11.9) |  |
| AFP (%) |  |  |  |  | 0.659 |
|  | ≤400ng/ml | 2036 (48.9) | 422 (47.3) | 440 (49.2) |  |
|  | >400ng/ml | 2130 (51.1) | 470 (52.7) | 454 (50.8) |  |
| Cirrhosis (%) |  |  |  |  | 0.739 |
|  | No | 1658 (39.8) | 364 (40.8) | 366 (40.9) |  |
|  | Yes | 2508 (60.2) | 528 (59.2) | 528 (59.1) |  |
|  | Cause-HBV | 2304 (91.9) † | 485 (91.9) † | 483 (91.5) † |  |
|  | Cause-Alcohol | 195 (7.8) † | 42 (8.0) † | 43 (8.1) † |  |
|  | Cause-other# | 9 (0.4) † | 1 (0.2) † | 2 (0.4) † |  |
| CSPH (%) |  |  |  |  | 0.454 |
|  | No | 3075 (73.8) | 661 (74.1) | 678 (75.8) |  |
|  | Yes | 1091 (26.2) | 231 (25.9) | 216 (24.2) |  |
| Child-Pugh (%) |  |  |  |  | 0.167 |
|  | A | 2502 (60.1) | 558 (62.6) | 560 (62.8) |  |
|  | B | 1664 (39.9) | 334 (37.4) | 332 (37.2) |  |
| PVTT (%) |  |  |  |  | 0.173 |
|  | Presence | 386 (9.3) | 80 (9.0) | 100 (11.2) |  |
|  | Absence | 3780 (90.7) | 812 (91.0) | 794 (88.8) |  |
| HBsAg (%) |  |  |  |  | 0.569 |
|  | No | 712 (17.1) | 156 (17.5) | 166 (18.6) |  |
|  | Yes | 3454 (82.9) | 736 (82.5) | 728 (81.4) |  |
| HCV* (%) |  |  |  |  | 0.467 |
|  | No | 4162 (99.9) | 890 (99.8) | 892 (99.8) |  |
|  | Yes | 4 (0.1) | 2 (0.2) | 2 (0.2) |  |
| Drink history (%) |  |  |  |  | 0.981 |
|  | No | 3156 (75.8) | 676 (75.8) | 680 (76.1) |  |
|  | Yes | 1010 (24.2) | 216 (24.2) | 214 (23.9) |  |
| Sarcopenia (%) |  |  |  |  | 0.578 |
|  | No | 2166 (52.0) | 468 (52.5) | 482 (53.9) |  |
|  | Yes | 2000 (48.0) | 424 (47.5) | 412 (46.1) |  |
| Hypertension (%) |  |  |  |  | 0.708 |
|  | No | 3211 (77.1) | 686 (76.9) | 700 (78.3) |  |
|  | Yes | 955 (22.9) | 206 (23.1) | 194 (21.7) |  |
| ALB (%) |  |  |  |  | 0.528 |
|  | ≤35g/L | 1832 (44.0) | 396 (44.4) | 376 (42.1) |  |
|  | >35g/L | 2334 (56.0) | 496 (55.6) | 518 (57.9) |  |
| ALBI grade (%) |  |  |  |  | 0.988 |
|  | 1 | 3804 (91.3) | 812 (91.0) | 812 (90.8) |  |
|  | 2 | 329 (7.9) | 72 (8.1) | 74 (8.3) |  |
|  | 3 | 33 (0.8) | 8 (0.9) | 8 (0.9) |  |
| Platelets (%) |  |  |  |  | 0.165 |
|  | ≤150/mm^3^ | 791 (19.0) | 151 (16.9) | 151 (16.9) |  |
|  | >150/mm^3^ | 3375 (81.0) | 741 (83.1) | 743 (83.1) |  |
| Creatinine (%) |  |  |  |  | 0.790 |
|  | ≤1.2 mg/dL | 4129 (99.1) | 883 (99.0) | 884 (98.9) |  |
|  | >1.2 mg/dL | 37 (0.9) | 9 (1.0) | 10 (1.1) |  |
| Bilirubin (%) |  |  |  |  | 0.756 |
|  | ≤1.2 mg/dL | 4074 (97.8) | 875 (98.1) | 877 (98.1) |  |
|  | >1.2 mg/dL | 92 (2.2) | 17 (1.9) | 17 (1.9) |  |

The values in parentheses are percentages unless indicated otherwise.

^‡^ χ^2^ test with Yates’ correction.

*Indicates the use of Fisher's exact test.

† indicates the proportion of patients with liver cirrhosis.

#"Other" refers to liver cirrhosis caused by autoimmune factors, genetic and metabolic diseases, fatty liver, and similar conditions.

Abbreviations: AFP: alpha-fetoprotein; ALB: albumin; BMI: Body Mass Index; CSPH: Clinically Significant Portal Hypertension; GGT: γ-glutamyl transpeptidase; HBsAg: hepatitis B surface antigen; HCC: hepatocellular carcinoma; HCV: Hepatitis C Virus; PVTT: Portal Vein Tumor Thrombus

**Supplementary Table 3. Risk factors for HCC rupture in Western Country**

| **1st author** | **Risk Factors** |
| --- | --- |
| Adham E Obeidat | BMI ≥ 30 (OR = 2.45, 95%CI: 1.21–4.97, P = 0.02);  Tumor size ≥ 5 cm (OR = 3.16, 95%CI: 1.66-6.02, P  <0.001);  Single tumors (OR = 2.08, 95%CI: 1.03–4.16, P = 0.030) |
|  |  |
| Gaëtan-Romain Joliat | Preoperative albumin level (OR=1.23, 95%CI: 1.11-1.32; P = 0.001);  Model for End-Stage Liver Disease (MELD) score (OR=1.08, 95%CI: 1.04-1.12; P = 0.003);  ASA grade (OR = 3.53, 95%CI: 1.64-7.61; P = 0.002); Child–Pugh grade (OR = 43.12, 95%CI: 1.52-1200.54; P = 0.027) |

Abbreviations: ASA: American Society of Anesthesiologists; BMI: Body Mass Index; CI: Confidence Interval; Child-Pugh: Child–Pugh Classification; HCC: Hepatocellular Carcinoma; MELD: Model for End-Stage Liver Disease; OR: Odds Ratio

**Supplementary Table 4. Evaluation indicators for each model**

| **Model** |  | **Precision** | **Recall** | **Accuracy** | **F1 score** | **ROC-AUC** | **95%CI** |
| --- | --- | --- | --- | --- | --- | --- | --- |
| CAPTure |  |  |  |  |  |  |  |
|  | Average 10-fold Training | 0.885 | 0.889 | 0.889 | 0.887 | 0.857 | 0.811-0.934 |
|  | Validation | 0.859 | 0.879 | 0.879 | 0.869 | 0.824 | 0.766-0.914 |
|  | Test | 0.860 | 0.855 | 0.855 | 0.857 | 0.840 | 0.791-0.922 |
| Random Forest |  |  |  |  |  |  |  |
|  | Average 10-fold Training | 0.879 | 0.880 | 0.880 | 0.880 | 0.899 | 0.835-0.949 |
|  | Validation | 0.865 | 0.881 | 0.881 | 0.873 | 0.809 | 0.706-0.912 |
|  | Test | 0.858 | 0.877 | 0.877 | 0.867 | 0.870 | 0.798-0.975 |
| Deep Learning |  |  |  |  |  |  |  |
|  | Average 10-fold Training | 0.877 | 0.866 | 0.890 | 0.871 | 0.943 | 0.874-0.992 |
|  | Validation | 0.869 | 0.861 | 0.867 | 0.865 | 0.928 | 0.845-0.971 |
|  | Test | 0.870 | 0.832 | 0.872 | 0.851 | 0.892 | 0.799-0.964 |

Abbreviation: ROC: receiver operator characteristic; AUC: area under the curve; CI: confidence interval.
